# Correction to: Study of Different Cultivated Plants Rhizosphere Soil Fungi-Mediated Pectinase: Insights into Production, Optimization, Purification, Biocompatibility, and Application

**DOI:** 10.1007/s00248-025-02498-0

**Published:** 2025-01-28

**Authors:** Mai Ali Mwaheb, Basant Mohamed Abd El-Aziz, Basma T. Abd-Elhalim, Nabil Abo El-Kassim, Tharwat E. E. Radwan

**Affiliations:** 1 Botany Department, Faculty of Science, Fayoum University, Fayoum, 63514 Egypt; 2 Department of Agricultural Microbiology, Faculty of Agriculture, Ain Shams University, Shubra El-Khaimah, 11241 Cairo Egypt

**Correction to: Microbial Ecology (2024) 87:165**

10.1007/s00248-024-02474-0

[Current corresponding author: Error]

The corresponding author name is in the recently published manuscript Basma Talaat Abd-Elhalim.

[Corrected corresponding author name: Erratum]

To be **Basma T. Abd-Elhalim.**

[Current abstract: Error]

The enzyme’s molecular weight was approximately 30 kDa.

[Corrected abstract: Erratum]

The enzyme’s molecular weight was approximately **35 kDa**.

"The original version has been corrected."

